# Genetic variability affects absolute and relative potencies and kinetics of the anesthetics isoflurane and sevoflurane in *Drosophila melanogaster*

**DOI:** 10.1038/s41598-018-20720-7

**Published:** 2018-02-05

**Authors:** Zachariah P. G. Olufs, Carin A. Loewen, Barry Ganetzky, David A. Wassarman, Misha Perouansky

**Affiliations:** 1 Department of Anesthesiology, School of Medicine and Public Health, University of Wisconsin-Madison, Madison, WI 53706 USA; 2 Department of Medical Genetics, School of Medicine and Public Health, University of Wisconsin-Madison, Madison, WI 53706 USA; 3 Department of Genetics, College of Agriculture and Life Sciences, University of Wisconsin-Madison, Madison, WI 53706 USA

## Abstract

Genetic variability affects the response to numerous xenobiotics but its role in the clinically-observed irregular responses to general anesthetics remains uncertain. To investigate the pharmacogenetics of volatile general anesthetics (VGAs), we developed a Serial Anesthesia Array apparatus to expose multiple *Drosophila melanogaster* samples to VGAs and behavioral assays to determine pharmacokinetic and pharmacodynamic properties of VGAs. We studied the VGAs isoflurane and sevoflurane in four wild type strains from the Drosophila Genetic Reference Panel, two commonly used laboratory strains (Canton S and *w*^*1118*^), and a mutant in Complex I of the mitochondrial electron transport chain (*ND23*^*60114*^). In all seven strains, isoflurane was more potent than sevoflurane, as predicted by their relative lipid solubilities, and emergence from isoflurane was slower than from sevoflurane, reproducing cardinal pharmacokinetic and pharmacodynamic properties in mammals. In addition, *ND23*^*60114*^ flies were more sensitive to both agents, as observed in worms, mice, and humans carrying Complex I mutations. Moreover, we found substantial variability among the fly strains both in absolute and in relative pharmacokinetic and pharmacodynamic profiles of isoflurane and sevoflurane. These data indicate that naturally occurring genetic variations measurably influence cardinal pharmacologic properties of VGAs and that flies can be used to identify relevant genetic variations.

## Introduction

Isoflurane (ISO) and sevoflurane (SEVO) are chemically closely related volatile general anesthetics (VGAs) with distinct properties^1,2^. Like all other VGAs, ISO and SEVO achieve the anesthetic phenotype within a very narrow concentration range^3^. However, deviations in susceptibility to anesthetics exist: inbred strains of rodents differ in sensitivity to ISO^4–6^ and enflurane^7^. Individual mongrel cats show differential sensitivity to modern VGAs^8^ as do segregated human populations for SEVO^9–11^. These data raise the possibility that anesthetic sensitivity may behave like a quantitative trait^12,13^, as opposed to being mediated by a small number of discrete molecular targets. By contrast, Mendelian mutations have pronounced effects on the sensitivity to VGAs in model organisms^14–16^ and can result in life-threatening consequences^17,18^. Therefore, as for other xenobiotics^19,20^, genetic polymorphisms that place a phenotypically normal individual at increased risk for adverse effects from exposure to VGAs are likely to exist.

To facilitate research into anesthetic pharmacogenomics, we took advantage of the genetically accessible fruit fly *Drosophila melanogaster*. Flies are not humans, yet evolution has produced cellular and molecular mechanisms that are conserved between flies and humans. For example, 55% to 75% of disease-causing genes in humans have functional homologues in flies^21^. Furthermore, because of evolutionary conservation, findings initially made in flies have made possible clinically important discoveries in higher organisms. Therefore, we used flies to establish the extent to which key pharmacokinetic (PK: ‘how an organism affects a drug’) and pharmacodynamic (PD: ‘how a drug affects an organism’) properties of ISO and SEVO are conserved in flies. This included testing whether a hypomorphic mutation in Complex I of the mitochondrial electron transport chain (ETC) reproduces the hypersensitive phenotype observed in *Caenorhabditis elegans*, *Mus musculus*, and *Homo sapiens*^22–25^ as well as examining the variability of PK and PD properties in a natural fly population, the Drosophila Genetic Reference Panel (DGRP).

Our data reveal substantial variability of PK and PD profiles for ISO and SEVO among genetically diverse wild type flies and support the usefulness of flies for pharmacogenomic and toxicological research.

## Results

### Design of the Serial Anesthesia Array

**Figure 1:**
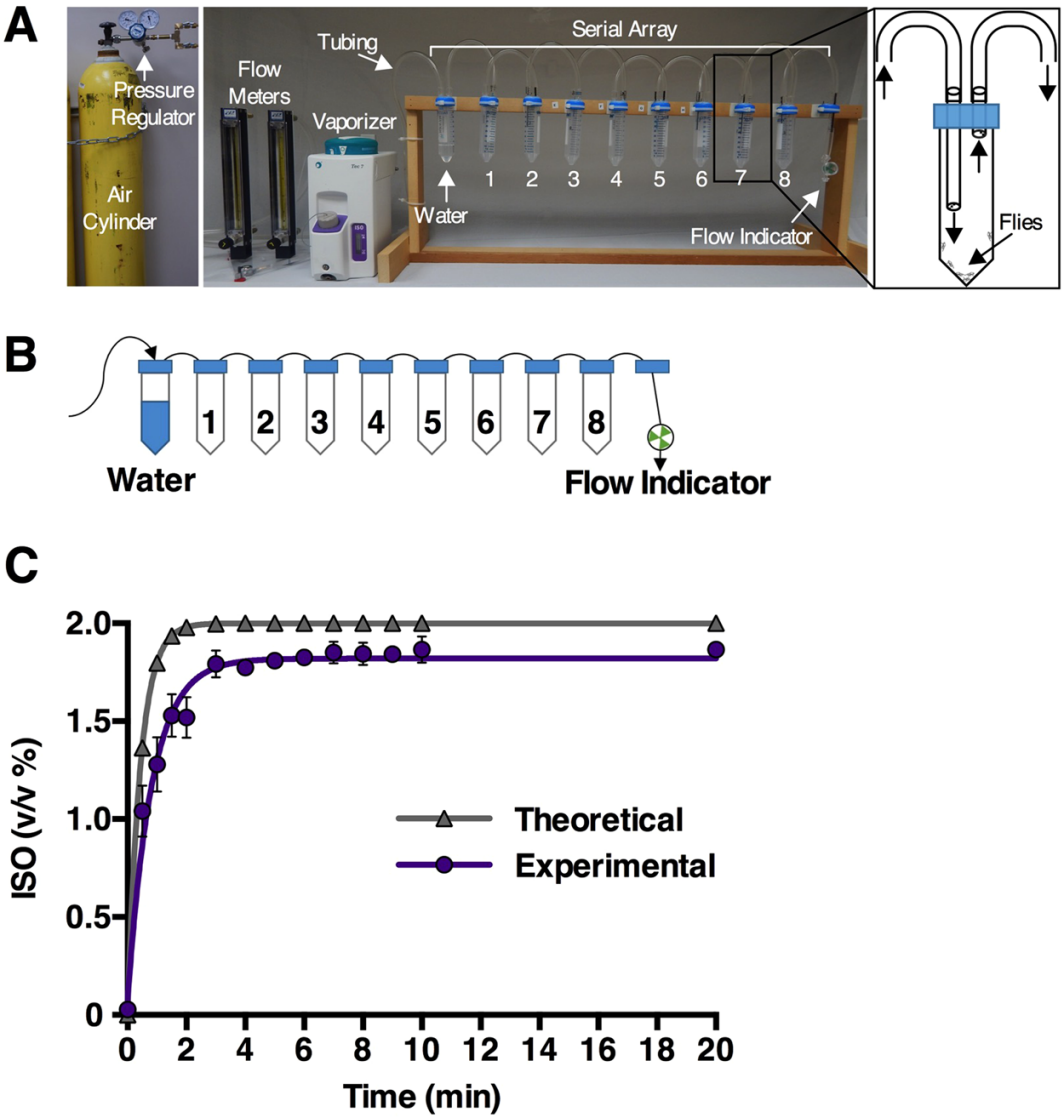
The Serial Anesthesia Array. (**A**) Main components of the array are an agent-specific anesthetic vaporizer, flow meters, and a serial array of anesthetizing positions. The small footprint allows housing within a conventional fume hood. A compressed air cylinder supplies air via a pressure regulator to the flow meter. The vaporizer controls the vaporization of liquid anesthetics and accurately delivers a given concentration to the air flowing from the air cylinder. Anesthetizing positions are assembled out of 50 mL conical tubes with customized caps fitted with input and output ports made of polystyrene pipettes. The tubes are interconnected via the external pipette ends with Tygon tubing. Within each tube the upstream (inflow) port opening is positioned closer to the bottom than the downstream (outflow) port to facilitate complete gas exchange (inset diagram). A fine mesh covers outflow ports to prevent flies from escaping. The number of positions in the array is flexible. The standard 8 + 1 configuration is shown. (**B**) The first position in the array contains water to humidify the mixture of air and VGA, and the remaining positions are available for experimental samples. A flow indicator at the downstream end of the array monitors the activity of the system. (**C**) Quantification of the rise in ISO concentration in the downstream tube 8 with air flowing at 1.5 L/min. Black circles indicate gas chromatograph measurements of ISO concentration in tube 8 over time (n ≥ 2, R^2^ = 0.95). Gray triangles indicate the theoretical change in anesthetic concentration calculated using the equation C_Array_ = C_AF_(1-e^−T/τ^) (see Materials and Methods for details), assuming a single compartment of equivalent volume.

To allow comparative studies of VGA pharmacology on a medium-throughput scale in Drosophila with different genetic backgrounds, we assembled the Serial Anesthesia Array from easily accessible components. In the configuration shown in Fig. 1A,B, the array has eight tube positions for experimental samples and one tube position for humidification of the carrier gas (8 + 1 configuration). The tubes are arranged in series with a total volume of 655 mL downstream of a vaporizer and an air cylinder. Each tube can hold thousands of flies, but we standardly used fewer than 100 flies per tube. The array provides a simple system with a small footprint capable of delivering any volatile agent with precise and flexible control of the dose (concentration X duration of exposure) simultaneously to multiple experimental samples, e.g. flies of different age, sex, or genotype. Furthermore, neither the concentration nor the duration of VGA administration is constrained by a behavioral endpoint (e.g. loss of postural control) that limits the dose of VGA that can be delivered in traditional inebriometer or separator devices^26–28^.

#### Gas exchange in the array is rapid

We tested the dynamics of anesthetic delivery through the array by measuring the rise in anesthetic concentration in the most downstream position (tube 8) (Fig. 1A,B). Analysis by gas chromatography showed that the ISO concentration rose with a time constant τ of 0.83 min (Fig. 1C). For a single compartment of the same volume (655 mL), the calculated τ would be 0.44 min. The slower than expected rise in ISO concentration is probably due to turbulence and inhomogeneity of gas exchange during the transition through nine separate tubes as opposed to uptake into flies whose biomass is negligible in comparison to the volume of the tubes. However, gas exchange was efficient enough to reach 95% of the set ISO concentration in the most downstream position within 2.5 min. We conclude that gas exchange in the array permits efficient and rapid administration of VGAs.

### Behavioral assays as measures of anesthetic PK and PD

We developed behavioral assays to characterize the PK and PD of VGAs. Like the Dispersion assay developed by Nash and colleagues^29^, our Early Recovery Time and Percent Active assays are based on the innate, startle-induced climbing behavior of Drosophila. Contrary to the passive assays employed in inebriometer or separator chambers, which use loss of resistance to gravity as a measure of anesthetic depth^30–32^, our assays rely on the integrated function of different behaviors, including the escape reflex in response to mechanical stress, negative geotaxis proper (an innate orienting response and movement in opposition to gravitational cues), climbing ability, and locomotor activity itself, thereby capturing a more complex behavior.

Early Recovery Time is a measure of the time to recovery from a standard VGA dose (2% ISO or 3.5% SEVO for 1 hr) that produces deep anesthesia. After venting the anesthetic from the vial, startle-induced climbing behavior was elicited by manually tapping the vial on a rubber pad to knock the flies to the bottom of the vial once per minute until 6 of 60 flies (10%) were able to climb above 2 cm in 10 sec. Unanesthetized flies easily met this criterion upon the first tap. Thus, Early Recovery Time captures the PK of initial emergence from anesthesia.

The Percent Active is the percentage of 10 flies that climb above 2 cm in 1 min after climbing is elicited by manually tapping the vial. We used the Percent Active assay to determine the half-maximal time to recovery (TtR_50_). This was done by exposing flies to standard VGA concentrations (2% ISO or 3.5% SEVO) for 1 hr followed by venting the anesthetic from the vial and repeating the Percent Active measurement in the absence of anesthetic until all flies met the climbing criterion (above 2 cm in 1 min). The TtR_50_ is the amount of time needed for 50% of the flies in a vial to climb above 2 cm in 1 min, and it is an indication of the PK of extensive recovery from anesthesia.

We also used the Percent Active assay to determine the half-maximal effective concentration (EC_50_) of a VGA. This was done by exposing flies to different anesthetic concentrations for 10 min, measuring the Percent Active in the presence of anesthetic, increasing the anesthetic concentration, and repeating the Percent Active measurement until no flies met the climbing criterion (above 2 cm in 1 min). We chose a 10 min exposure to ensure complete equilibration of the fly brain with the VGA, which is estimated to take approximately 1 min^33,34^. The EC_50_ is an indication of the PD of induction of anesthesia.

### The Serial Anesthesia Array delivers equivalent concentrations of anesthetic to all positions

**Figure 2:**
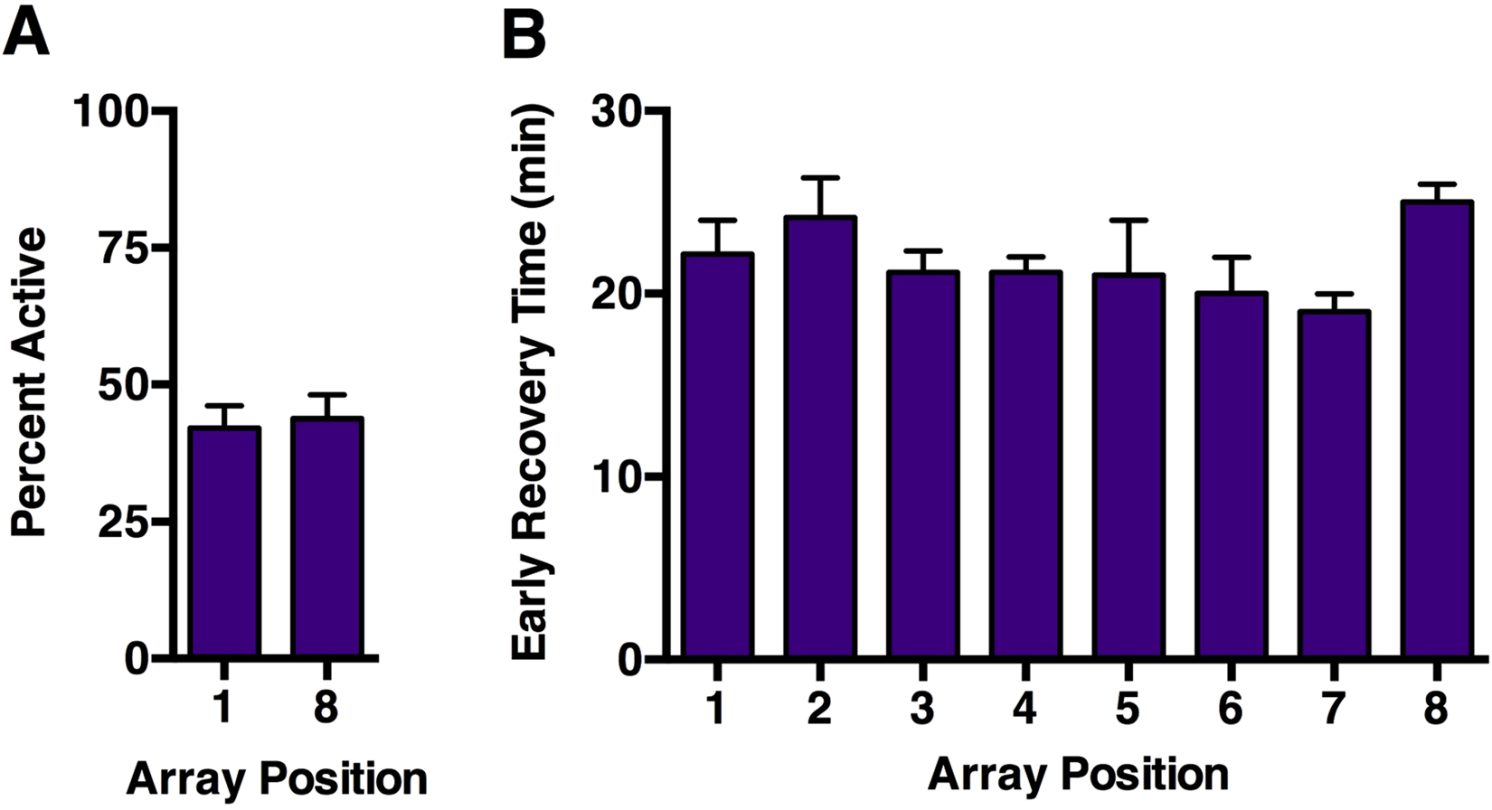
All positions in the Serial Anesthesia Array receive an equivalent concentration of VGA. (**A**) The Percent Active of male *w*^*1118*^ flies exposed to 0.4% ISO at 1–8 days of age for 10 min in the most upstream (tube 1) and downstream (tube 8) positions (n = 9) (P = 0.77, unpaired equal variance two-tail t-test). (**B**) The Early Recovery Time of mixed sex *w*^*1118*^ flies exposed to 2% ISO for 1 h at 0–7 days of age in tubes 1–8 (n = 2) (P = 0.36, one-way ANOVA).

We designed the Serial Anesthesia Array to compare anesthetic PK and PD on a medium-throughput experimental scale among different VGAs and different experimental samples. For the latter goal, it is important that all positions arranged in series in the array yield comparable experimental conditions. Therefore, we performed a functional characterization of the array by comparing Percent Active and Early Recovery Time measurements of *w*^*1118*^ flies (a standard laboratory strain) in different positions of the array. We found that the Percent Active did not differ between male flies exposed to 0.4% ISO for 10 min in the most upstream position (tube 1) and the most downstream position (tube 8) (42.09 ± 4.02 and 43.83 ± 4.31, respectively, Fig. 2A). Similarly, Early Recovery Time of mixed sex *w*^*1118*^ flies ranged from 19.0 ± 1.0 min to 25.0 ± 1.0 min but did not differ systematically among positions in the array (P = 0.36, one-way ANOVA) (Fig. 2B). In summary, these results indicate that the Serial Anesthesia Array rapidly delivers equivalent doses of anesthetic to flies in all positions, thereby making it possible to study the effects of anesthetics on multiple fly samples at the same time.

#### Sex but not age affects the PK properties of VGAs

**Figure 3:**
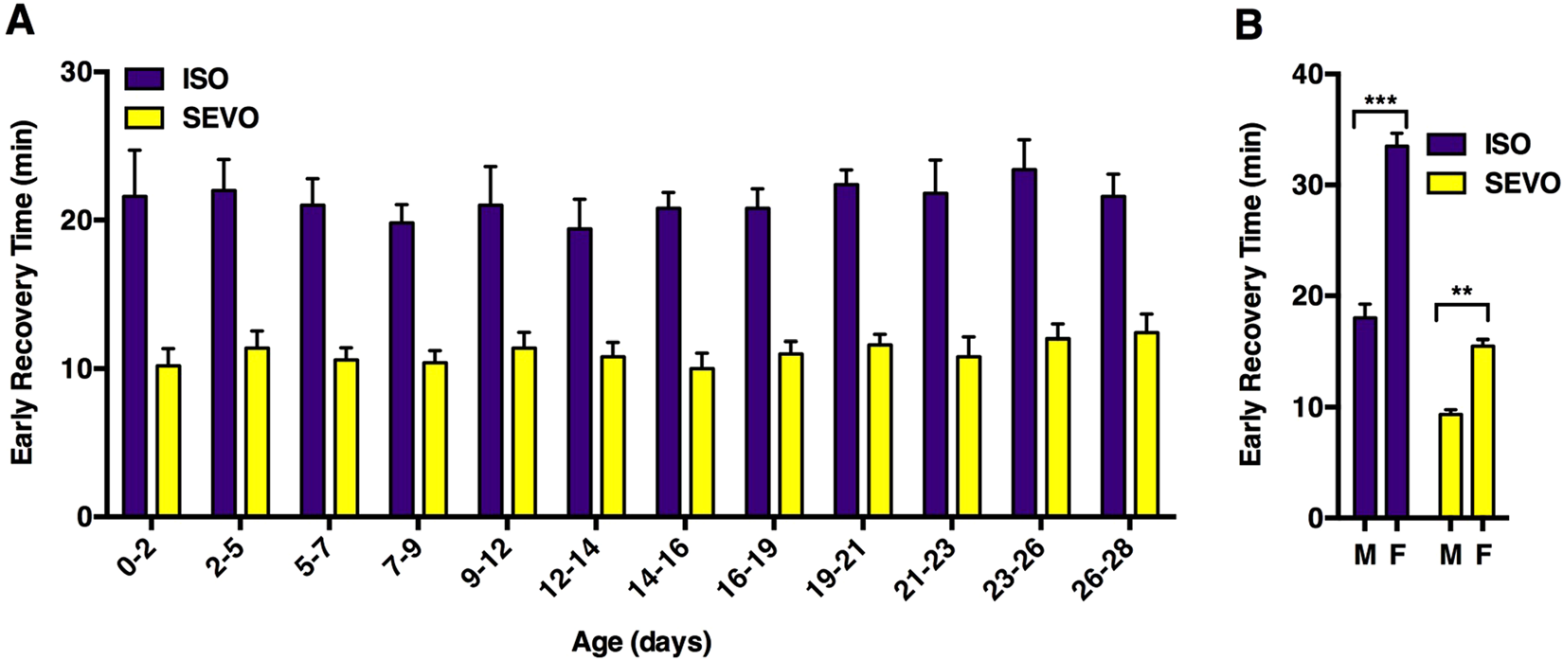
Sex but not age influence the speed of recovery from anesthesia. (**A**) The Early Recovery Time of mixed sex *w*^*1118*^ flies exposed to anesthesia at the indicated age ranges (n = 5) (ISO P = 0.98 and SEVO P = 0.89, one-way ANOVA). (**B**) The Early Recovery Time of *w*^*1118*^ male (M) and female (F) flies exposed to anesthesia at 1–8 days old (n = 3) (**P < 0.01, ***P < 0.001, unpaired equal variance two-tail t-test).

To test whether age at the time of exposure influences recovery from deep anesthesia, we measured Early Recovery Time in mixed sex *w*^*1118*^ flies grouped in 3-day age increments from 0–2 to 26–28 days old. At all ages, recovery was faster from SEVO than ISO, but age did not affect the speed of recovery from either anesthetic (ISO P = 0.98 and SEVO P = 0.89, one-way ANOVA) (Fig. 3A). Hence, as in humans, the PK of these agents is stable in adulthood^35^. However, sex did affect the recovery from both anesthetics. We found that 1–7 day old *w*^*1118*^ and Canton S (two standard wild type strains) males recovered about twice as fast as females (Fig. 3B and Supplemental Fig. 1).

#### Deep anesthesia does not affect lifespan

**Figure 4:**
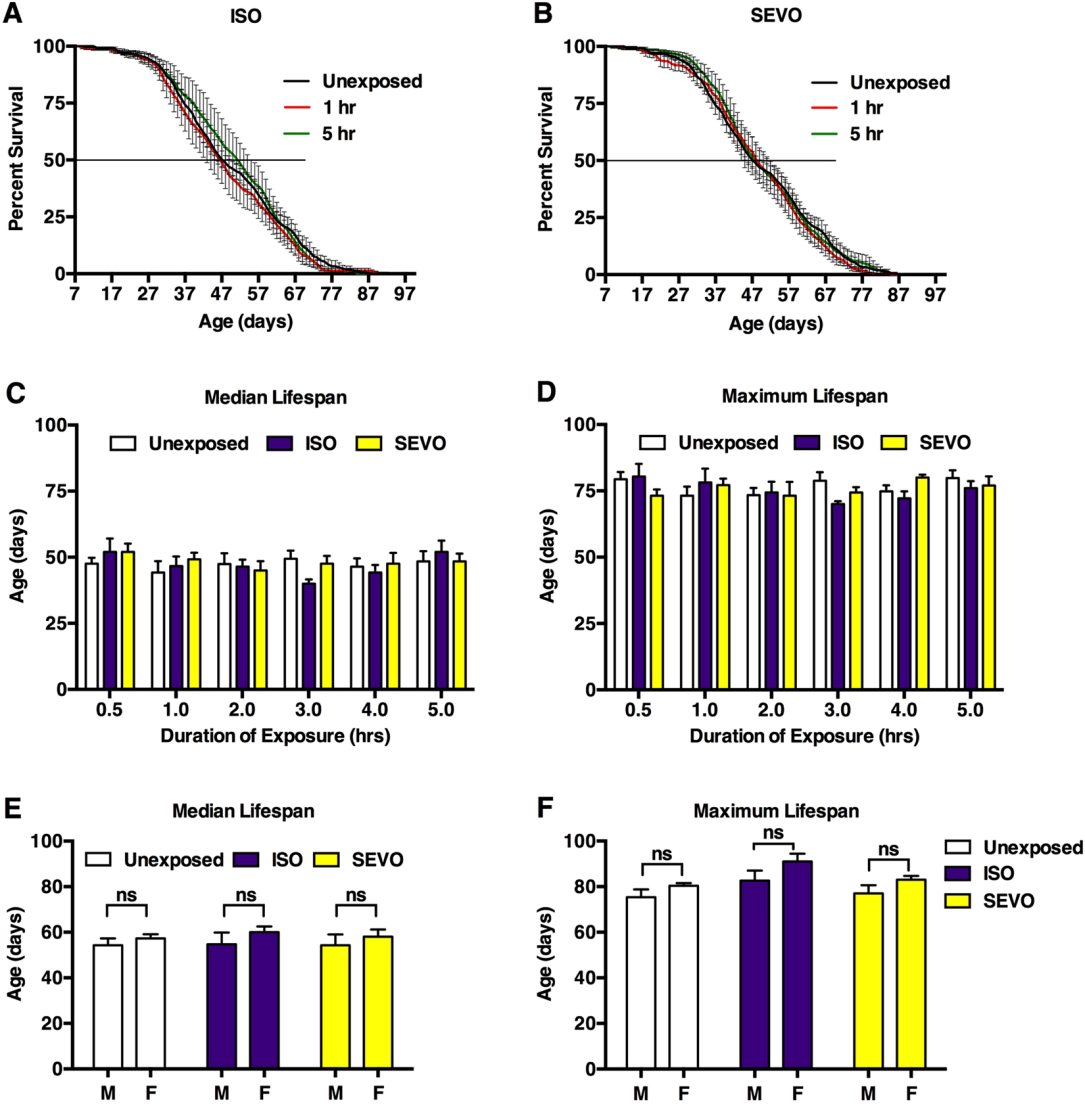
Deep anesthesia does not affect lifespan. Survival curves for mixed sex *w*^*1118*^ flies exposed to (**A**) 2% ISO (264–289 flies) or (**B**) 3.5% SEVO (264–285 flies) for 1 hr (red) or 5 hr (green) at 0–7 days old. (**C**) Median and (**D**) maximum lifespans for exposure times from 0.5 to 5.0 hr (n = 5). (**E**) Median and (**F**) maximum lifespans for 1–8 day old males and female flies exposed to 2% ISO or 3.5% SEVO for 2 hr (n = 3) (not significant (ns), unpaired equal variance two-tail t-test).

We tested whether administration of anesthesia to mixed sex *w*^*1118*^ flies at 0–7 days old affected lifespan. After exposures ranging from 0.5 to 5 hr to either 2% ISO or 3.5% SEVO the percentage of surviving flies was determined until all flies had died (Fig. 4A,B and Supplemental Figs 2–4). Neither the duration of exposure nor the anesthetic agent affected the median or maximum lifespan (Fig. 4C,D). Furthermore, exposure of 1–8 day old male or female *w*^*1118*^ flies to 2% ISO or 3.5% SEVO for 2 hr had no effect on the median or maximum lifespan (Fig. 4E,F and Supplemental Fig. 4). We conclude that, as in rodents^36^, a single exposure to VGAs does not influence lifespan in healthy flies.

#### PK and PD properties of VGA in flies are similar to mammals

Studies comparing the potency of VGAs using the immobility-based minimum alveolar concentration of anesthetic (MAC) concept, which is assumed to reflect the concentration in the central nervous system, yield remarkably consistent results across all mammalian species and all VGAs^3^. MAC relies on a binary score (purposeful limb movement: yes or no) scaled to deep levels of anesthesia that is insensitive to quantitative nuances. More important, in mammals, MAC readout is determined by drug action in the spinal cord as opposed to the brain^37,38^. Therefore, applying the classic definition of MAC to invertebrates is problematic due to the difficulty of translating this classic endpoint to organisms with alternative behavioral repertoires^39^. Hence, researchers have developed a variety of species-specific behavioral endpoints to position experiments in invertebrates into pharmacologically meaningful contexts with studies in mammals. These endpoint definitions have resulted in a wide range of reported EC_50_s even for a single agent and a single fly strain, e.g. the EC_50_s for ISO in Canton S was 0.36 ± 0.04 v/v % in our hands and ranged from 0.20% to 0.71% in the literature^40,41^.

**Figure 5:**
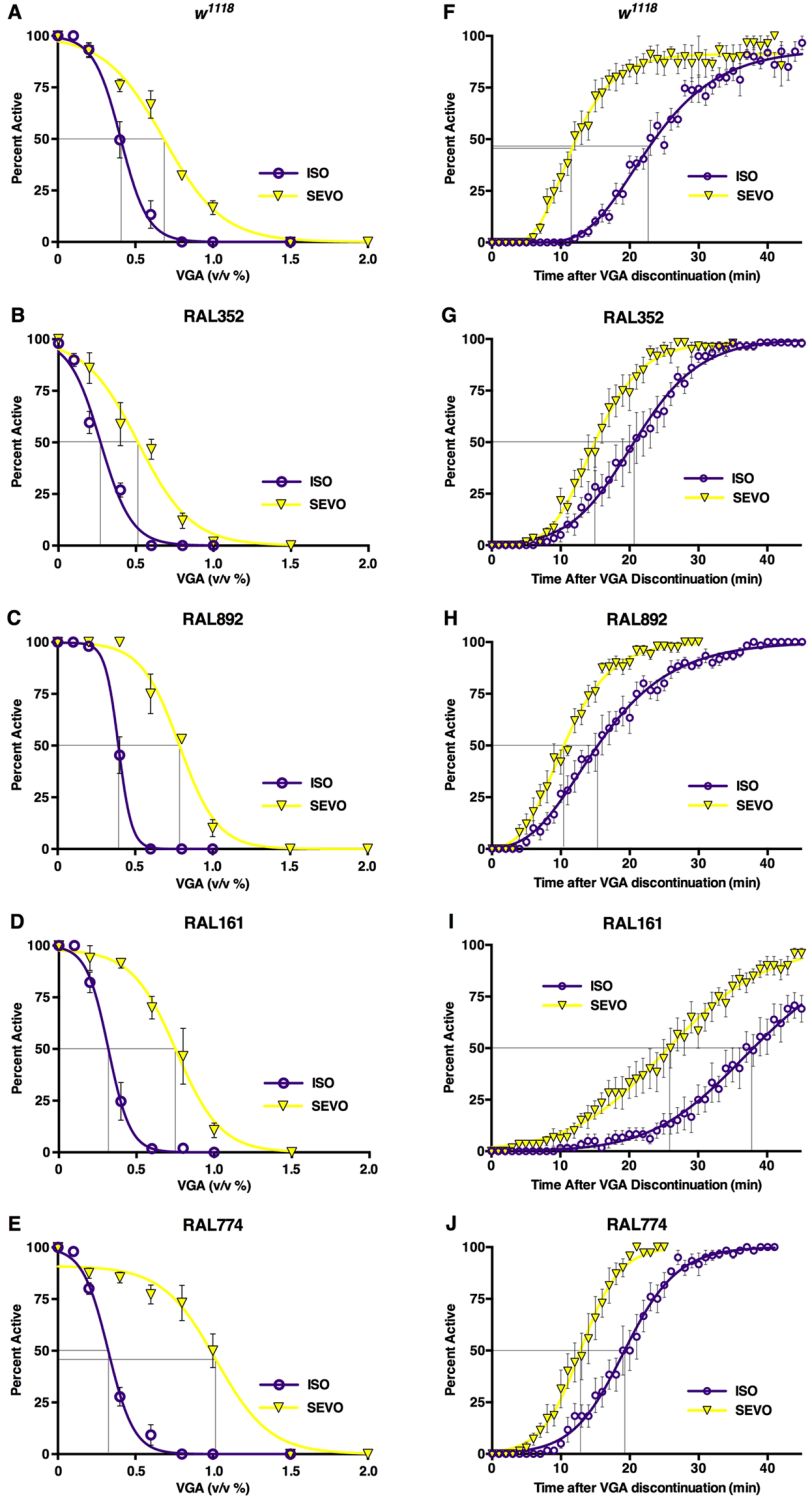
Genetic background modulates the PK and PD properties of VGAs. Panels A-E: Dose-response relationships for ISO and SEVO used to calculate EC_50_s. The Percent Active was measured in 1–8 day-old male flies at different concentrations (*n* = 3–5 per genotype and agent). Panels F-J: Time course of recovery from anesthesia (2% ISO or 3.5% SEVO for 1 hr) for the same genotypes as in panels A-E. (n = 3–7 per genotype and agent).

To determine the extent to which clinically important PK and PD properties of VGAs are reproducible in flies, we used the Percent Active assay to compare anesthetic dynamics of ISO and SEVO in *w*^*1118*^ flies. Because sex affects PK properties (Fig. 3B), only males were used for these studies. In 1–7 day old *w*^*1118*^ males, ISO was more potent than SEVO (EC_50_: 0.41 ± 0.03 v/v % and 0.68 ± 0.05 v/v %, respectively) with a potency ratio (ISO/SEVO) of 0.60 (0.41/0.68) (Fig. 5A and Table 1). Recovery from deep anesthesia was significantly faster for SEVO than for ISO (TtR_50_: 11.66 ± 0.77 min and 22.94 ± 1.0 min, respectively) with a recovery ratio (ISO/SEVO) of 1.97 (22.94/11.66) (Fig. 5F and Table 1). The potency and recovery differences between ISO and SEVO in flies are consistent with differences observed in humans^42^.

**Table 1:** Measures of anesthetic potency (EC_50_) and speed of recovery (TtR_50_) for isoflurane (ISO) and sevoflurane (SEVO).

Strain	EC_50_ (v/v %)		Potency Ratio (ISO/SEVO)	TtR_50_ (min)		Recovery Ratio (ISO/SEVO)
	ISO	SEVO		ISO	SEVO	
*w* ^*1118*^	0.41 ± 0.03	0.68 ± 0.05	0.60 ± 0.05	22.94 ± 1.00	11.66 ± 0.77	1.97 ± 0.16
RAL352	0.27 ± 0.05	0.51 ± 0.11	0.53 ± 0.14	20.68 ± 0.93	14.91 ± 0.66	1.39 ± 0.09
RAL892	0.39 ± 0.02	0.79 ± 0.04	0.49 ± 0.04	15.37 ± 0.79	10.46 ± 0.46	1.47 ± 0.10
*ND23* ^*60114*^	0.20 ± 0.01	0.47 ± 0.03	0.42 ± 0.04	ND	ND	ND
RAL161	0.32 ± 0.04	0.76 ± 0.07	0.42 ± 0.06	37.86 ± 1.26	25.71 ± 1.96	1.47 ± 0.12
Canton S	0.36 ± 0.04	0.89 ± 0.11	0.40 ± 0.07	22.49 ± 1.33	17.59 ± 1.49	1.28 ± 0.13
RAL774	0.33 ± 0.03	1.02 ± 0.08	0.32 ± 0.04	19.25 ± 0.72	12.86 ± 0.92	1.50 ± 0.12

Strains are ordered based on the potency ratio (from high to low). EC_50_ and TtR_50_ shown as mean ± SD, propagation of error-corrected for ratios.

#### A mitochondrial mutation increases the potency of VGAs

**Figure 6:**
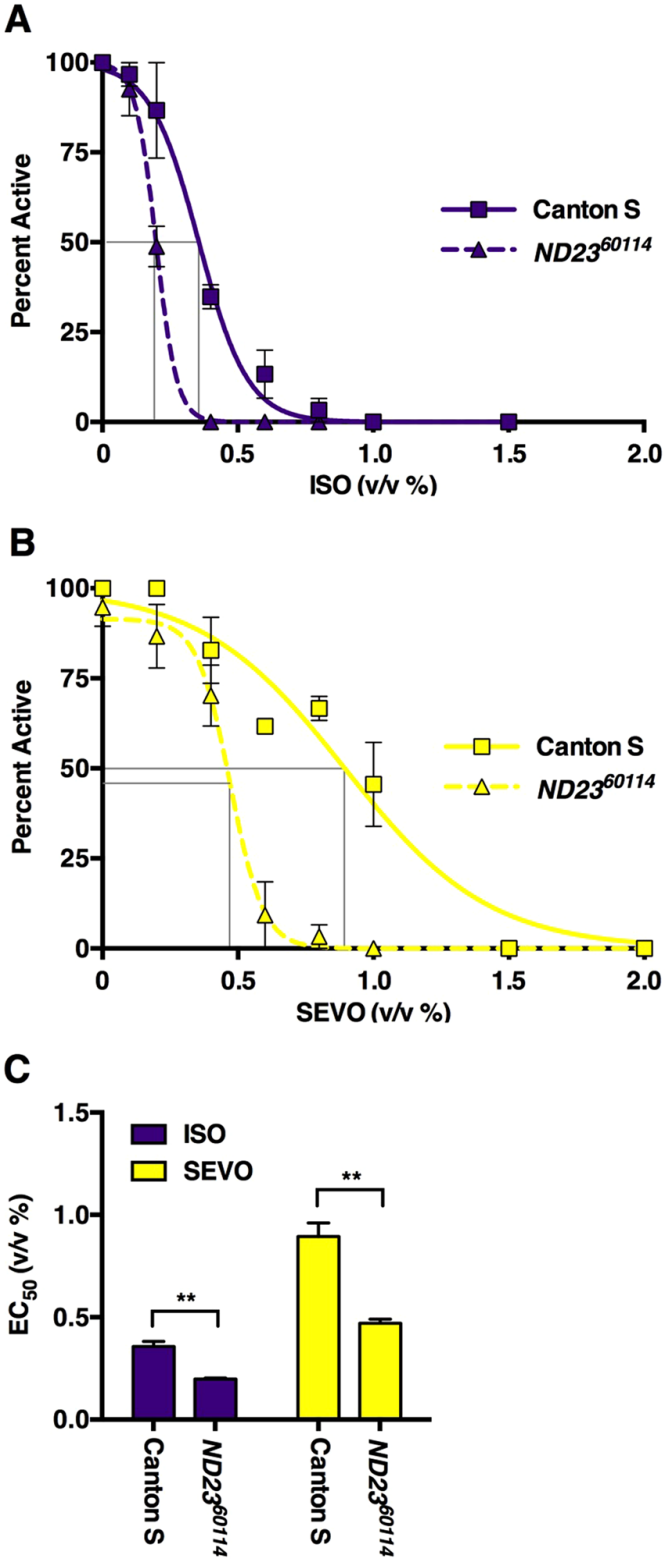
The *ND23*^*60114*^ mutation in Complex I of the mitochondrial electron transport chain increases the potency of ISO and SEVO. The Percent Active of 2–4 day old male Canton S and *ND23*^*60114*^ flies exposed to different concentration of (**A**) ISO (n = 3) or (**B**) SEVO (n = 3). (**C**) EC_50_s for Canton S and *ND23*^*60114*^ flies, derived from panels A and B, respectively (**P < 0.01, equal variance unpaired two-tail test).

One of the potential uses of the Serial Anesthesia Array is to identify heritable genetic variations that affect responses to anesthesia. To explore this possibility, we characterized the behavioral responses of *ND23* mutant flies to ISO and SEVO. *ND23* is a nuclear gene that encodes a core subunit of Complex I of the mitochondrial electron transport chain. Mutations in subunits of eukaryotic Complex 1, which contains at least 45 distinct subunits, lead to increased sensitivity to VGAs in *C. elegans*^22,23,43^, mice^24^ and humans^25^; however, the effects of mutations in ND23 (known as T20H4.5 in *C. elegans* and NDUFS8 in humans) on anesthetic sensitivity have not been studied in any organism. To assess whether the *ND23*^*60114*^ mutation sensitizes flies to anesthetics, we determined the EC_50_ for ISO and SEVO in 2–4 day old male flies. For both VGAs, the EC_50_ of *ND23*^*60114*^ flies was lower than that of its control background strain Canton S as well as *w*^*1118*^ (Fig. 6 and Table 1). We conclude that ND23 confers resistance to VGAs.

#### Genomic variation influences the PD and PK properties of VGAs

**Figure 7:**
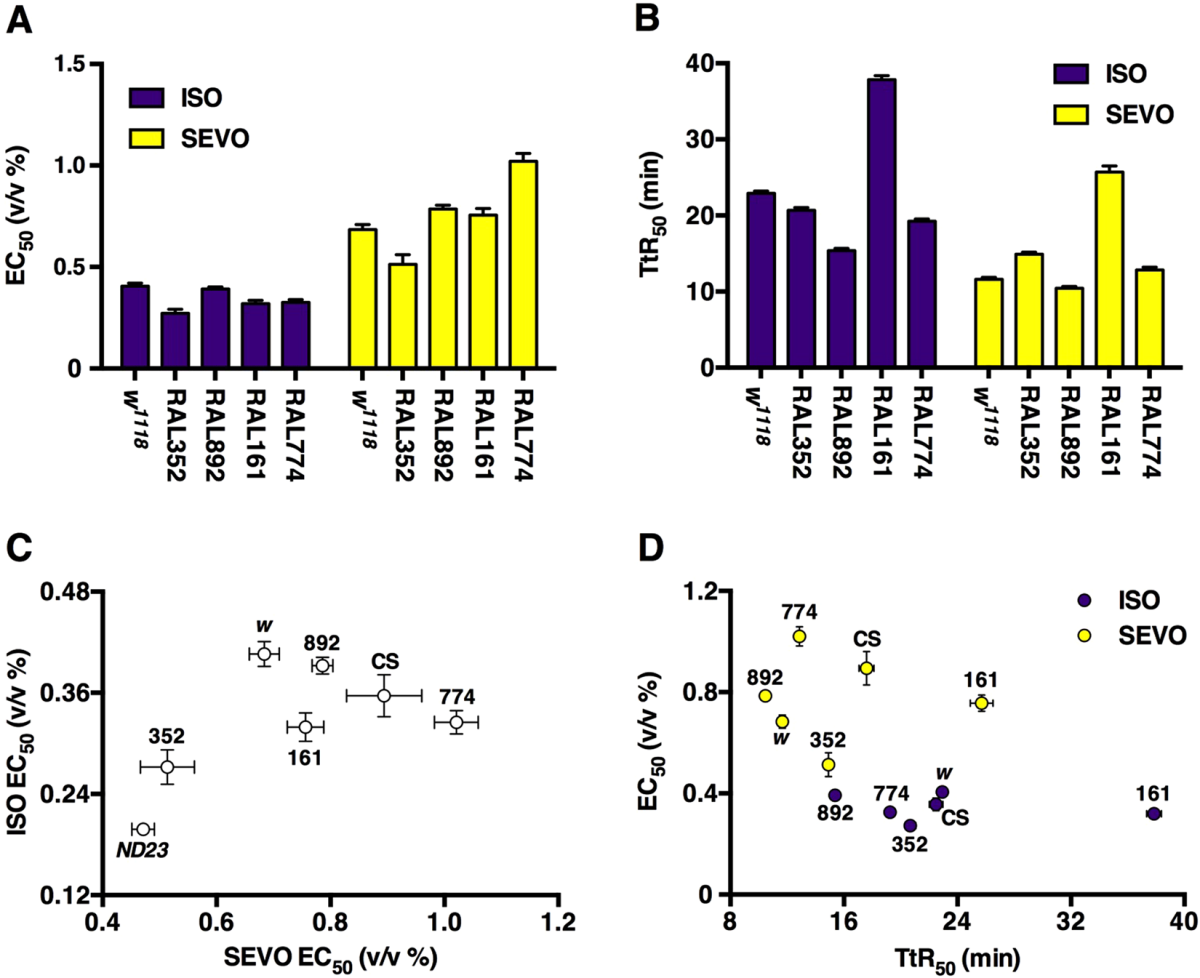
Natural Drosophila populations harbor a variety of responses to VGAs. Summary of EC_50_s (**A**) and TtR_50_s (**B**) from all tested fly lines (Figs 5 and 6) indicates that PD and PK vary unpredictably. (**C**) The EC_50_ values of one agent (ISO or SEVO) is a poor predictor of the EC_50_ of the other (R^2^ = 0.34). (**D**) The potency of an agent (ISO or SEVO) does not correlate with its rate of emergence: R^2^ = 0.069 and 0.001 for ISO and SEVO, respectively. Data are derived from experiments shown in Figs 5 and 6 and Supplemental Fig. 6 (see Table 1 for numerical values).

To explore the pharmacogenomics of VGAs in natural populations we determined the potency and the recovery profiles for ISO and SEVO in two standard laboratory strains (*w*^*1118*^ and Canton S) and four strains derived from a natural population (RAL161, 352, 774, 892). The specific RAL lines were chosen from opposite ends of the mortality spectrum from blunt trauma determined in 179 RAL lines^44^ from the Drosophila Genetic Reference Panel (DGRP)^45^: RAL352 and 774 are at the low end, while RAL161 and 892 are at the high end of the mortality spectrum^44^. In all RAL lines, ISO was more potent than SEVO (Fig. 5, Table 1). However, their potency ratios (ISO EC_50_/SEVO EC_50_) varied from 0.32 ± 0.04 (RAL774) to 0.53 ± 0.14 (RAL352) (Table 1). No correlation between the previously determined blunt trauma resilience and sensitivity to VGAs was discernible. *ND23*^*60114*^ was more sensitive to VGAs than all other lines tested: the ISO EC_50_ of the most sensitive tested RAL line (RAL352) was 35% higher than that of *ND23*^*60114*^ and the EC_50_ of SEVO was 9% higher. The potency ratios (ISO/SEVO) of all seven lines ranged from 0.32 ± 0.04 (RAL774) to 0.60 ± 0.05 (*w*^*1118*^) (Fig. 7A and Table 1). These results indicate that natural and laboratory populations harbor genetic polymorphisms that differentially modify the behavioral response to chemically closely-related VGAs.

We also examined the extent to which genetic variation influences recovery from VGAs, a PK parameter not previously examined in invertebrate model organisms. In RAL lines, the ISO TtR_50_s ranged from 15.37 ± 0.79 min (RAL892) to 37.86 ± 1.26 min (RAL161) and the TtR_50_s for SEVO ranged from 10.46 ± 0.46 min (RAL892) to 25.71 ± 1.96 min (RAL161) (Figs 5F–J and 7B, and Table 1). As in *w*^*1118*^ flies, recovery from SEVO was faster than from ISO in all tested RAL lines, but the absolute speed of recovery and relative speed of recovery (i.e., ISO TtR_50_/SEVO TtR_50_) were different among the four lines (Table 1). These results indicate that both PD and PK profiles (reflected in the EC_50_ and TtR_50_, respectively) of VGAs are influenced by genetic background.

## Discussion

We have modified existing experimental systems for studying VGAs in flies with the goal of efficiently comparing the effect profiles of VGAs, including PD and PK measures, across different genotypes and experimental interventions. The Serial Anesthesia Array administers identical anesthetic concentrations to multiple samples (Fig. 1A,B) with fast equilibration in all positions (Fig. 1C), assuring equal anesthetic exposure of all samples (Fig. 2). Each position in the array accommodates large numbers of flies providing robust measures for the condition under study. Furthermore, the array offers the ability to simultaneously examine different variables such as age and genotype under the exact same anesthetic dose (Figs 4–7). Using this system, we obtained robust and reproducible results that demonstrate that genetic background significantly modulates the responses to VGAs in flies.

The relative potencies of VGAs are key factors determining their use in clinical practice. We developed the Percent Active assay because reproducing MAC (the standard assay for potency in mammals^46^) in invertebrate model organisms is laborious, indirect and operator-dependent^47–51^. Using the Percent Active assay, we compared VGA potency across multiple fly lines (Figs 5–7 and Table 1). The resulting EC_50_s for ISO are comparable to those determined using analogous assays^33,34,40^, confirming the robustness of the fly model across laboratories. Furthermore, variability in the ISO/SEVO potency and recovery ratios among fly lines demonstrates that natural populations harbor a diversity of genetic polymorphisms that differentially modulate the potency of and the recovery from two prototypical VGAs (Table 1). In this context, it is notable that the potency ratio of the mitochondrial Complex I mutant *ND23*^*60114*^ was very close to that of its background strain Canton S despite substantially higher sensitivity (Fig. 6. and Table 1). This finding indicates that reduced activity of the electron transport chain proportionally affects the sensitivity to ISO and SEVO.

In addition to potency, the rate of recovery from anesthesia is a critical characteristic of all general anesthetics. We are not aware of any studies addressing recovery from VGAs in invertebrates. Therefore, we developed two behavioral assays, Early Recovery Time and Percent Active, to determine the PK of recovery from anesthesia in flies. Both assays demonstrated substantially faster recovery from SEVO compared with ISO replicating the situation in humans (Figs 3, 5, and 7; Supplemental Fig. 6; and Table 1)^42^. To compare recovery times among strains we use the ISO/SEVO recovery ratio derived from time-to-recovery measurements (Table 1). Its value varied considerably from 1.28 ± 0.13 (Canton S) to 1.97 ± 0.16 (*w*^*1118*^) bracketing the recovery ratio of 1.63 determined in Sprague-Dawley rats^52^. The range of absolute and relative potencies and recovery rates determined in RAL lines illustrate the degree to which variability in naturally occurring genetic architecture influences anesthetic PK and PD.

Recovery from VGAs is determined by the rate of elimination of the agent from the body, which occurs via the lungs in mammals and by diffusion out of tracheae, which is independent of circulation, in flies. As both ISO and SEVO undergo minimal metabolism in mammals, enzyme polymorphisms (the primary cause of differences in PK for the majority of xenobiotics)^19^ are unlikely to account for differences in the TtR_50_ that we observed. To reconcile our comparative findings for ISO and SEVO with standard understanding of VGA PK and PD, we consider the following possibilities. If diffusion out of the body alone determined the speed of recovery, ISO/SEVO TtR_50_ ratios should be similar among strains (which are all of similar size). However, we found that TtR_50_ ratios varied considerably (Table 1). Alternatively, if recovery was simply following declining drug concentrations at the target site, the degree of recovery from an agent should lag in a ‘sensitive’ strain (i.e. lower EC_50_) behind that in a ‘resistant’ strain (i.e. high EC_50_) because at equal brain concentration of agent a higher fraction of sensitive animals would be anesthetized. If this was the case, the EC_50_ would inversely correlate with TtR_50_. However, we found no correlation between the EC_50_ and the TtR_50_ (Fig. 7D). Lastly, a difference in brain dynamics may account for the lack of correlation between the EC_50_ and TtR_50_. The EC_50_ captures the transition from awake to anesthetized state, while the TtR_50_ quantifies the transition from anesthetized to awake state. Experiments with VGAs in flies and in mice demonstrate that the directionality of change in brain states, that is loss vs. recovery of function, occur at different points of a dose-response relationship^33^. Furthermore, the points differ among VGAs^33^ and, as suggested by our experiments, may differ among ‘wildtype’ strains. Together, these data indicate that genetic background influences the responsiveness to anesthetics in multiple ways.

Hypomorphic mutations in Complex I of the mitochondrial electron transport chain result in hypersensitivity to VGAs in humans^25^, mice^53^, and worms^23^. To the best of our knowledge, *ND23*^*60114*^ is the first description of a mutation in the electron transport chain affecting a phenotype under anesthesia in flies. *ND23* is an ortholog of *NDUFS8* in mammals and *T20H4.5* in *C. elegans*. In humans, mutations in *NDUFS8* are associated with Leigh syndrome, a progressive neurological degenerative disorder with onset in infancy or childhood^54^. RNAi knockdown of *T20H4.5* renders worms sensitive to halothane-induced immobilization, reducing the EC_50_ from 3.2% to 2.1%^55^. For the majority of mutations tested for VGA responsiveness in flies and worms, an understanding of the gene products and regulatory pathways affected is largely incomplete. However, mutations in core subunits of Complex I of the electron transport chain consistently result in varying degrees of hypersensitivity to most VGAs in worms^22,43,55^ and our data suggest that the same is true in flies. This finding indicates that a common molecular nexus located in mitochondria influences the potency of halothane, ISO, and SEVO and possibly other potent modern inhalational agents. However, mutants with increased anesthetic sensitivity unrelated to mitochondrial proteins have also been described^31,56^. So, while a research focus on mitochondrial genes is warranted, it is unlikely to completely explain open questions such as why there is a lack of correlation between the EC_50_ and TtR_50_.

In summary, genetic variability in the response to VGAs is relevant because phenotypically silent polymorphisms may directly modify not only the dose of anesthetics needed for surgery but also the likelihood of an individual experiencing the deleterious long-term collateral effects of VGAs (acute cognitive disorders, accelerated neurodegeneration, alterations in immune and inflammatory functions). The latter aspect may be more important as it may affect clinically relevant outcomes in surgical populations. So far, genomic modifiers of VGA sensitivity have been largely neglected in clinical studies examining potential long-term sequelae of exposure to VGAs. We expect that newly developed methods for the identification of quantitative trait loci in flies^45,57,58^, which have already been successfully applied to xenobiotic toxicology^59^, will contribute to bridging anesthetic phenotype to genotype.

## Materials and Methods

### Fly lines and culturing

All flies were maintained on standard molasses food containing 30 g Difco granulated agar (Becton-Dickinson, Sparks, MD), 44 g YSC-1 yeast (Sigma, St. Louis, MO), 328 g cornmeal (Lab Scientific, Highlands, NJ), 400 ml unsulphured Grandma’s molasses (Lab Scientific), 3.6 L water, 40 ml propionic acid (Sigma), and tegosept (8 g Methyl 4-hydroxybenzoate in 75 ml 95% ethanol) (Sigma). DGRP strains were obtained from the Bloomington Stock Center. The *ND23*^*60114*^ line is an EMS mutant line from the Ganetzky lab temperature-sensitive paralytic collection. Mix sex fly samples contained males and females in a 1:1 ratio (Supplemental Fig. 5). *w*^*1118*^ and DGRP flies were maintained at 25 °C. In Fig. 6, Canton S and *ND23*^*60114*^ flies were maintained at 25 °C and then switched to 29 °C at 0–2 days post-eclosion for the remainder of the experiment. In Supplemental Figs 1 and 6, Canton S flies were maintained at 25 °C.

#### Serial Anesthesia Array assembly, operation, and characterization

The Serial Anesthesia Array was constructed using Falcon 50 mL conical tubes (Fisher Scientific, Waltham, MA), polystyrene serological pipettes (Fisher Scientific), Tygon tubing AAC00022 5/16″ ID × 7/16″ OD (Saint-Gobain North America, Malvern, PA), a Bel-Art paddle style flow indicator model 19937-0003 (Cole-Parmer, Vernon Hills, IL), and calibrated, agent-specific Tec 7 vaporizers (Datex-Ohmeda, Inc., Madison, WI). ISO and SEVO were obtained from Piramal Enterprises Ltd. (Maharashtra, India). Additional details about assembly and operation of the array are provided in Fig. 1A,B and the Results section. A GOW-MAC Series 580 flame ionization gas chromatograph (GOW-MAC Instrument Co., Bethlehem, PA) calibrated with 1% ISO (Calibration cylinder, Air Liquide Healthcare, Madison, WI) was used to measure ISO concentrations. The time constant (τ) for ISO was calculated using the equation C_Array_ = C_AF_ (1-e^−T/τ^), where C_Array_ is the anesthetic concentration in the array, C_AF_ is the anesthetic concentration flowing into the array, T is sampling time after initiation of air flow, and τ is the time constant [τ = (capacity of the array in mL)/(flow rate mL/min)]. Using this equation and gas chromatograph measurements yielded the experimental time constant of the array (Fig. 1C).

#### Behavioral and lifespan assays

Effects of anesthetics on flies were quantified using customized, negative geotaxis-based behavioral Early Recovery Time and Percent Active assays, described in the Results section. To determine median and maximum lifespans after exposure to 2% ISO or 3.5% SEVO for various times, flies were cultured on molasses food at 25 °C and transferred to fresh food every 3–4 days. The number of dead flies was recorded daily (except weekends) until all flies had died. Additional details are provided in Fig. 4 and Supplemental Figs 2–4.

### Data analysis and statistics

We analyzed data using GraphPad Prism 6 software (La Jolla, CA) and calculated statistical significance between two data distributions using the unpaired equal-variance two-tail *t*-test. We used ANOVA for three or more data points. Error bars in all figures represent the standard error of the mean. Standard deviations are presented in Table 1.

## Electronic supplementary material

Supplementary figures
