# Supplementary material for: Genetic variability affects absolute and relative potencies and kinetics of the anesthetics isoflurane and sevoflurane in Drosophila melanogaster
Source: Sci Rep. 2018 Feb 5;8:2348. doi: 10.1038/s41598-018-20720-7 (PMC5799260; doi:10.1038/s41598-018-20720-7)
Supplement: Supplementary file 1 — Supplementary figures [file 41598_2018_20720_MOESM1_ESM.pdf]

Supplementary material

Genetic variability affects absolute and relative potencies and kinetics of the anesthetics isoflurane and sevoflurane in *Drosophila melanogaster*

Short title: Genetic background and anesthetic sensitivity

Zachariah P. G. Olufs[1, 2], Carin A. Loewen[3], Barry Ganetzky[3], David A. Wassarman[2], and Misha Perouansky[1,4]

[1]Department of Anesthesiology, School of Medicine and Public Health, University of Wisconsin-Madison, Madison, WI 53706

[2]Department of Medical Genetics, School of Medicine and Public Health, University of Wisconsin-Madison, Madison, WI 53706

[3]Department of Genetics, College of Agriculture and Life Sciences, University of Wisconsin-Madison, Madison, WI 53706

[4]Corresponding author

Misha Perouansky

Tel: 608-262-2903

E-mail: mperouansky@wisc.edu

Supplemental Figure 1

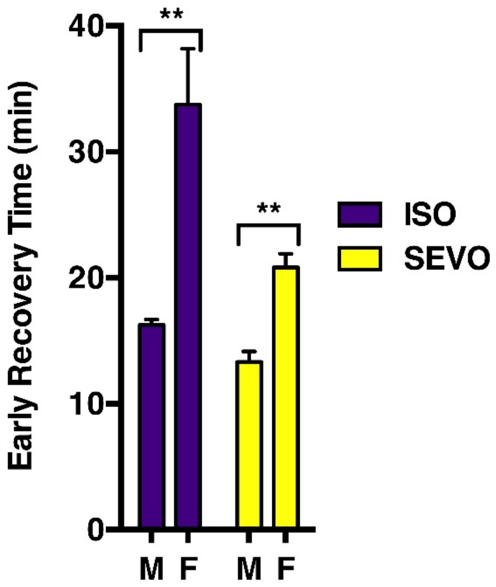

Sex influences the speed of recovery from anesthesia. The Early Recovery Time of Canton S male (M) and female (F) flies exposed to anesthesia at 1-8 days old (n≥3) (**P<0.01, unpaired equal variance two-tail t-test).

Supplemental Figure 2

Survival curves for mixed sex 0-7 day old w^{1118} flies exposed to (A) no anesthetic (264-289 flies), (B) 2% ISO (280-290 flies), and (C) 3.5% SEVO (282-287 flies) for the indicated times. Horizontal lines indicate the median lifespan. Supplemental Figures 2 and 3 present the same data grouped in different ways.

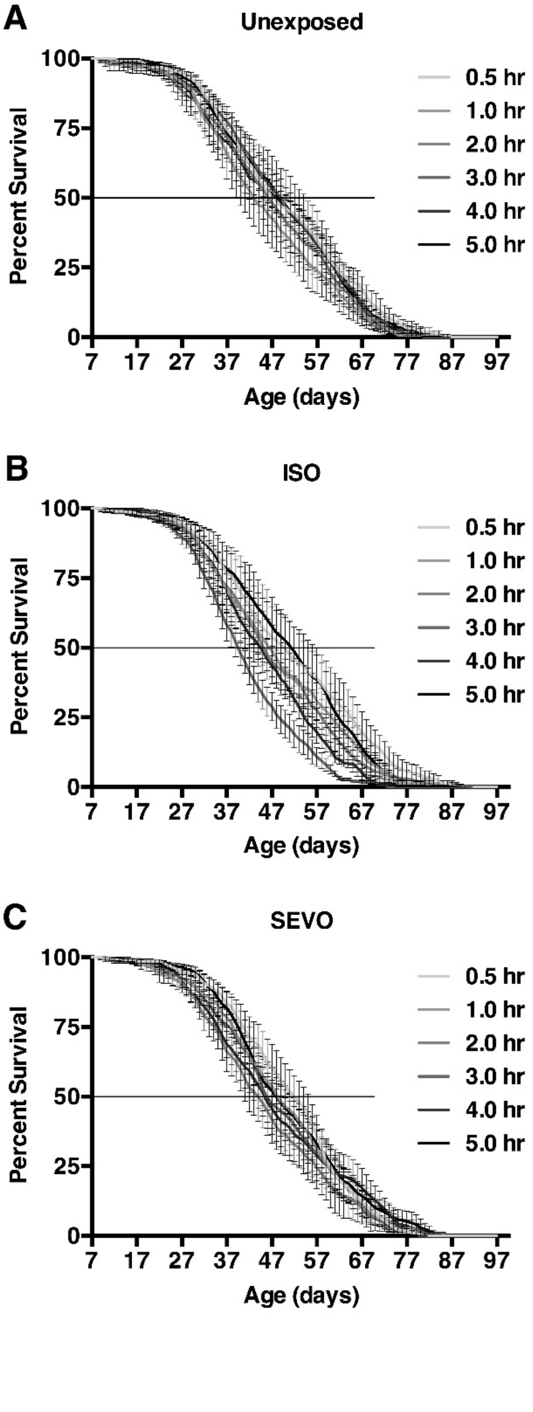

Supplemental Figure 3

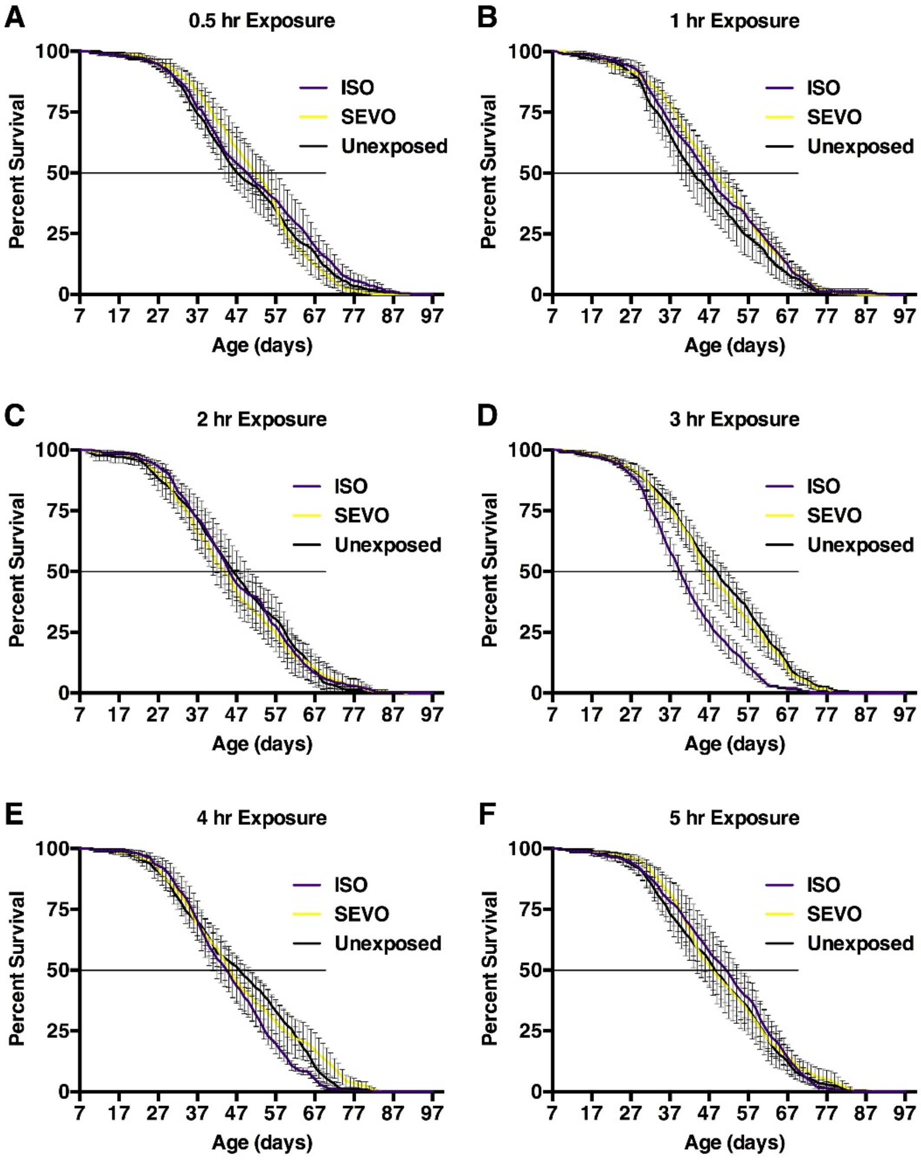

Survival curves for mixed sex 0-7 day old w^{1118} flies not exposed to anesthetic or exposed to 2% ISO or 3.5% SEVO for (A) 30 min (264-290 flies), (B) 1 hr (282-289 flies), (C) 2 hr (280-286 flies), (D) 3 hr (278-285 flies), (E) 4 hr (283-288 flies), and (F) 5 hr (266-281 flies). Horizontal lines indicate the median lifespan. Supplemental Figures 2 and 3 present the same data grouped in different ways.

Supplemental Figure 4

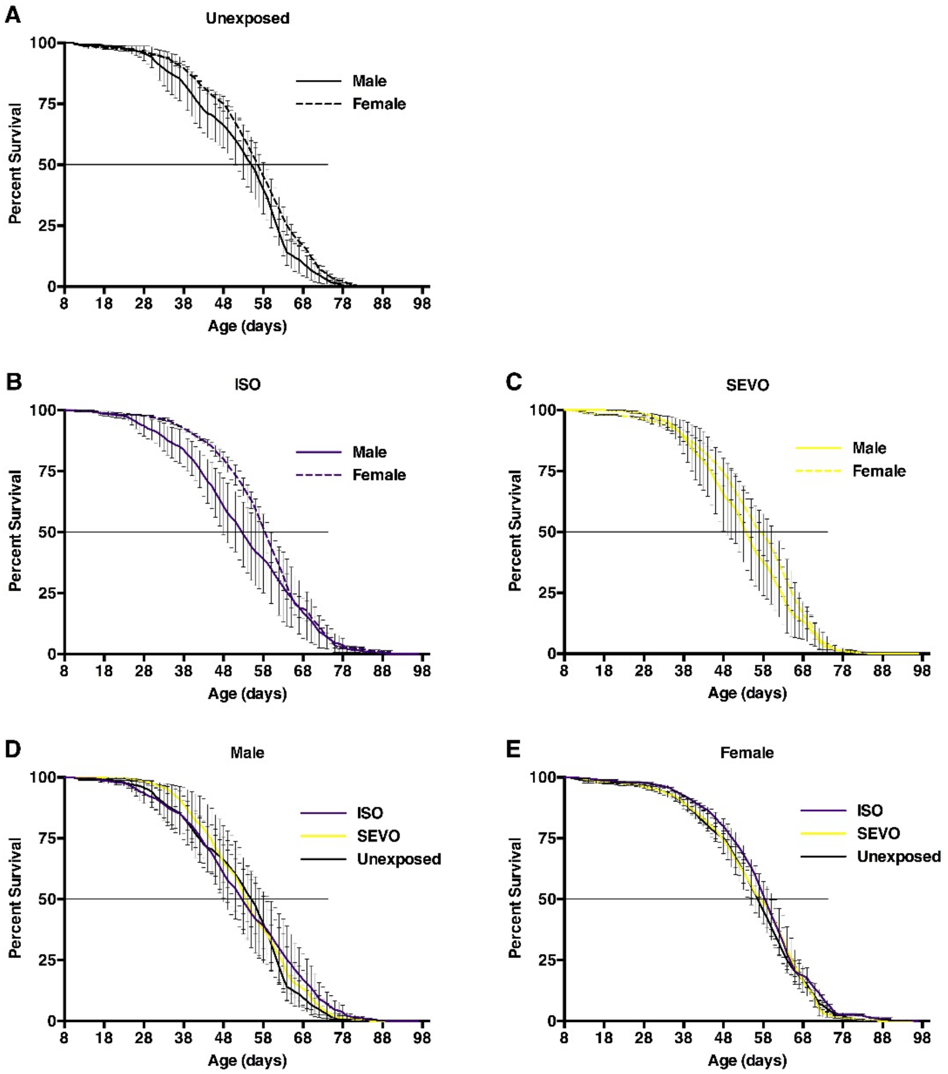

Survival curves for (A) 0-7 day old male and female w^{1118} flies not exposed to anesthetic (340-356 flies), (B) 0-7 day old male and female w^{1118} flies exposed to 2% ISO for 2 hr (338-349 flies), (C) 0-7 day old male and female w^{1118} flies exposed to 3.5% SEVO for 2 hr (347-350 flies), (D) 0-7 day old male w^{1118} flies not exposed to anesthetic or exposed to 2% ISO or 3.5% SEVO for 2 hr (338-347 flies), and (E) 0-7 day old female w^{1118} flies not exposed to anesthetic or exposed to 2% ISO or 3.5% SEVO for 2 hr (349-356 flies). Horizontal lines indicate the median lifespan.

Supplemental Figure 5.

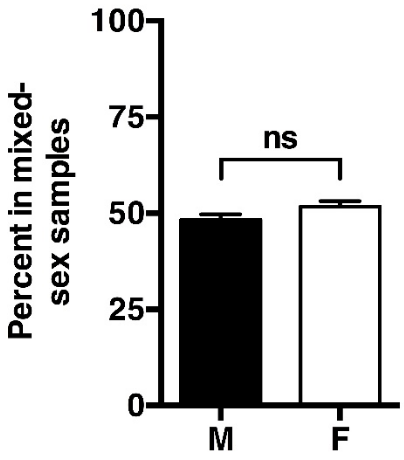

Males and females are equally represented in studies of mixed sex flies. The percentage of males and females in mixed sex samples (n=5) (1527 total flies, 738 males and 789 females) (P=0.74, unpaired equal variance two-tail t-test).

Supplemental Figure 6

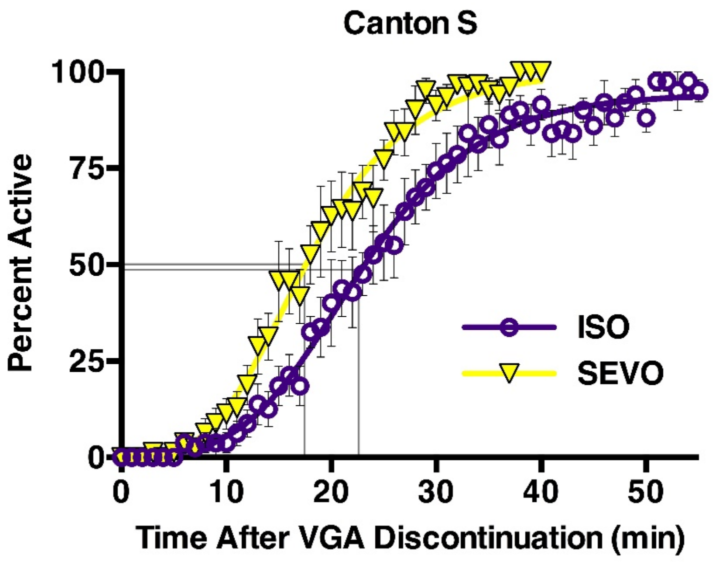

The PD properties of VGAs in Canton S flies. The Percent Active of 1-8 day old male Canton S flies over time after exposure to 2% ISO or 3.5% SEVO for 1 hr (n=8). TtR_{50} values derived from these data are presented in Figure 7D and Table 1.
